# A Wearable EMG-Driven Closed-Loop TENS Platform for Real-Time, Personalized Pain Modulation

**DOI:** 10.3390/s25165113

**Published:** 2025-08-18

**Authors:** Jiahao Du, Shengli Luo, Ping Shi

**Affiliations:** Institute of Rehabilitation Engineering and Technology, University of Shanghai for Science and Technology, Shanghai 200093, China; dujh@usst.edu.cn (J.D.); lsl24835@usst.edu.cn (S.L.)

**Keywords:** closed-loop neurostimulation, multi-channel current stimulation, real-time biosignal processing, surface electromyography, transcutaneous electrical nerve stimulation, wearable medical electronics

## Abstract

**Highlights:**

**What are the main findings?**

- Development of a wearable closed-loop TENS system: The proposed system integrates real-time sEMG acquisition, adaptive signal processing, and a boost-regulated power supply, enabling personalized, responsive neuromodulation.
- System validation with low-latency and stable output: The system demonstrates a closed-loop latency of less than 10 ms, stable biphasic waveforms (±22 mA) under prolonged operation, and minimal inter-channel leakage (<1.2%).

**What is the implication of the main finding?**

- Real-time, ambulatory neuromodulation: The system provides a platform for personalized, mobile neuromodulation, offering potential for non-invasive pain management and motor rehabilitation.
- Enhanced clinical feasibility: The low-latency and reliable multi-channel output could pave the way for future clinical applications, such as biofeedback-based therapies and neurorehabilitation.

**Abstract:**

A wearable closed-loop transcutaneous electrical nerve stimulation (TENS) platform has been developed to address the limitations of conventional open-loop neuromodulation systems. Unlike existing systems such as CLoSES—which targets intracranial stimulation—and electromyography-triggered functional electrical stimulation (EMG-FES) platforms primarily used for motor rehabilitation, the proposed device uniquely integrates low-latency surface electromyography (sEMG)-driven control with six-channel current stimulation in a fully wearable, non-invasive format aimed at ambulatory pain modulation. The system combines real-time sEMG acquisition, adaptive signal processing, a programmable multi-channel stimulation engine, and a high-voltage, boost-regulated power supply within a compact, battery-powered architecture. Bench-top evaluations demonstrate rapid response to EMG events and stable biphasic output (±22 mA) across all channels with high electrical isolation. A human-subject protocol using the Cold Pressor Test (CPT), heart rate variability (HRV), and galvanic skin response (GSR) has been designed to evaluate analgesic efficacy. While institutional review board (IRB) approval is pending, the system establishes a robust foundation for future personalized, mobile neuromodulation therapies.

## 1. Introduction

Transcutaneous electrical nerve stimulation (TENS) is a widely adopted non-invasive technique for alleviating both acute and chronic pain [1]. It functions by delivering low-frequency electrical pulses to peripheral nerves through surface electrodes, thereby modulating nociceptive pathways without the use of pharmacological agents [2]. With the advancement of wearable healthcare technologies, TENS has found increasing application in clinical settings such as musculoskeletal disorders [3], neuropathic pain [4], and functional rehabilitation [5]. However, the majority of commercially available TENS devices employ open-loop architectures with fixed stimulation parameters, limiting their ability to adapt to real-time physiological fluctuations and individual-specific variations [6]. This lack of adaptability often results in neural habituation, reduced therapeutic efficacy, and inconsistent clinical outcomes. Furthermore, generating the high-voltage output typically required for effective nerve activation—often exceeding 80 V—while adhering to the power and dimensional constraints of wearable hardware presents a persistent engineering challenge.

Recent developments have aimed to improve the portability, usability, and functional flexibility of TENS systems through features such as smartphone-based control [7], pulse-width modulation techniques [8], and high-compliance voltage drivers. These advancements have contributed to enhanced user convenience and stimulation precision, with some designs incorporating compact boost converters capable of delivering over 90 V from single-cell battery sources [9]. Additionally, wireless communication and mobile applications have enabled remote adjustment of stimulation parameters [10]. Despite these improvements, most systems continue to rely on open-loop configurations and single-site stimulation, lacking integration of biosignal feedback and adaptive control strategies. Moreover, very few platforms successfully combine real-time electromyography (sEMG)-driven control, programmable multi-channel output, and energy-efficient high-voltage regulation in a wearable format. Consequently, the full potential of TENS as a personalized, responsive neuromodulation modality remains largely unrealized.

While prior closed-loop neuromodulation platforms such as CLoSES [11] have demonstrated adaptive control in invasive intracranial applications, and EMG-triggered functional electrical stimulation (EMG-FES) systems [12] have been used in motor rehabilitation, these systems are either non-wearable, limited to motor control, or exhibit high latency. To date, no solution has integrated low-latency, multi-channel TENS stimulation driven by surface EMG into a compact, wearable format specifically designed for real-time, ambulatory pain modulation. To contextualize the novelty of our system, key distinctions from prior closed-loop neuromodulation platforms are summarized in Table 1, including differences in architecture, biosignal input, application domain, and latency performance. As summarized in Table 1, key distinctions from previous closed-loop platforms are presented to contextualize the novelty of our system.

To address these limitations, a wearable closed-loop TENS platform responsive to sEMG signals has been developed to enable real-time, personalized neuromodulation. The proposed system integrates real-time sEMG acquisition, adaptive signal processing, multi-channel programmable stimulation, and a PI-controlled boost converter capable of delivering up to 100 V, all within a compact, battery-powered architecture. A closed-loop feedback mechanism dynamically adjusts stimulation parameters in response to muscle activity, eliminating the need for manual calibration. System functionality has been validated through benchtop electrical tests. Additionally, a prospective human-subject study protocol has been designed, incorporating Cold Pressor Testing to assess analgesic efficacy, with trials pending ethical approval. The remainder of this paper is organized as follows: Section 2 details the system architecture and hardware implementation; Section 3 presents experimental methods and results; Section 4 discusses key findings and implications; and Section 5 concludes the work.

## 2. System Design and Implementation

**Figure 1:**
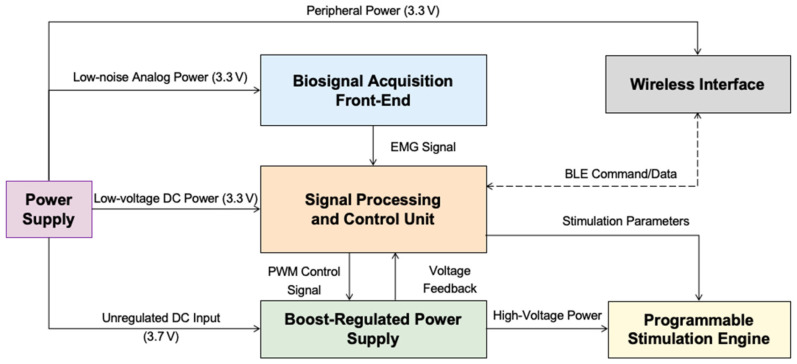
Block diagram of the wearable EMG-driven closed-loop TENS system. The architecture includes four primary modules: a biosignal acquisition front-end, a signal processing and control unit, a programmable stimulation engine, and a boost-regulated power supply. The central controller performs real-time EMG signal processing, adjusts stimulation parameters, and regulates high-voltage output using a PI-controlled boost converter. The entire system operates from a compact, battery-based power source with multiple voltage domains, including low-noise analog and peripheral rails. Wireless communication is implemented via a bidirectional BLE interface. The design prioritizes modularity, signal integrity, and power efficiency to support real-time, ambulatory neuromodulation.

The proposed system is a fully integrated, wearable platform designed to deliver TENS under closed-loop control driven by sEMG signals. As illustrated in Figure 1, the system comprises four primary modules: a biosignal acquisition front-end, a signal processing and control unit, a programmable stimulation engine, and a boost-regulated power supply. These components are implemented on a compact, battery-powered embedded system featuring wireless connectivity and a mobile interface. The overall design prioritizes modularity, scalability, and power efficiency to enable real-time operation in ambulatory environments.

### 2.1. Biosignal Acquisition Front-End

The biosignal acquisition module is designed to capture sEMG signals from targeted muscle groups using disposable Ag/AgCl electrodes. A two-stage amplification and filtering circuit is employed to ensure high signal fidelity [13]. The first stage consists of an instrumentation amplifier (AD8232) with a gain of 100 *v*/*v* and a high common-mode rejection ratio (CMRR > 80 dB) to suppress ambient noise [14].This is followed by an active bandpass filter with a low cutoff at 20 Hz and a high cutoff at 500 Hz, implemented using a second-order Sallen-Key topology, which isolates the sEMG frequency range while attenuating motion artifacts and power-line interference [15]. The analog front-end is fabricated on a four-layer PCB featuring separated analog and digital ground planes, star-grounding for noise suppression, and tight analog trace routing to minimize cross-talk. The conditioned signal is then digitized using a 12-bit analog-to-digital converter (ADC) sampling at 1 kHz, which provides adequate temporal resolution for real-time processing. The analog front-end is implemented with low-noise, ultra-low-power components optimized for wearable applications.

### 2.2. Signal Processing and Control Unit

The digitized sEMG signals are processed by a control unit implemented on a low-power STM32-based microcontroller unit (MCU). This unit performs real-time signal conditioning, event detection, and closed-loop control. Initially, a moving average filter is applied to suppress high-frequency noise [16]. Voluntary muscle activations are then detected using a threshold-based algorithm [17,18]. To evaluate detection performance, we tested the threshold-based sEMG trigger on 10 recorded sessions from two users, each performing repeated wrist extensions under low-noise conditions. Sensitivity and specificity were computed against ground-truth annotated onsets. The resulting receiver operating characteristic (ROC) curve yielded an area under the curve (AUC) of 0.94. At the optimal threshold, the system achieved a sensitivity of 92.6% and a specificity of 90.3%. These values indicate strong discriminative ability across user and trial variability (see Table 2). Based on the extracted sEMG activity, stimulation parameters—such as pulse width and amplitude—are dynamically adjusted. The MCU also coordinates stimulation timing and manages Bluetooth Low Energy (BLE) communication with the user interface. The control algorithm is optimized for low latency and minimal memory overhead to support efficient embedded deployment in portable neuromodulation systems.

To improve system robustness during dynamic or ambulatory use, several enhancements are planned for future iterations. These include adaptive thresholding based on real-time baseline estimation to account for gradual signal drift and user movement. Additionally, signal quality monitoring techniques—such as short-term energy stability and signal-to-noise ratio (SNR) tracking—will be explored to identify conditions such as electrode detachment or excessive artifacts. These indicators can serve as inputs for automatic re-calibration routines or prompt real-time user feedback via the interface.

### 2.3. Boost-Regulated Power Supply

To provide high-voltage output for the stimulation engine, a proportional–integral (PI) controlled boost converter is integrated into the system. The proportional gain (Kp) and integral gain (Ki) of the controller are critical for balancing the response speed and the degree of overshoot during dynamic load changes. Given the absence of detailed experimental data for tuning these values, the Kp = 0.15 and Ki = 0.02 parameters were chosen based on standard control system design practices, aiming to achieve a fast system response while minimizing voltage overshoot. A higher Kp would typically reduce the settling time, but could result in overshoot, while increasing Ki could reduce steady-state error, but at the cost of potentially increasing system oscillations. The values of Kp and Ki were selected to minimize voltage fluctuations, with the target voltage kept within ±2.5 V of the desired output, while ensuring that the system response time remains under 30 ms. We anticipate further refinement of these parameters based on experimental validation. The converter steps up the 3.7 V battery input to a programmable output of up to 100 V. Output voltage is monitored via a resistive voltage divider, which supplies feedback to the microcontroller for closed-loop regulation [19]. Pulse-width modulation (PWM) is employed to stabilize the output under varying load conditions. The power module incorporates thermal protection and electromagnetic shielding to reduce electromagnetic interference (EMI) while maximizing energy efficiency. Although high-voltage boost architectures are commonly used in neuromodulation systems, few implementations provide integrated voltage regulation within such a compact embedded platform [20].

### 2.4. Programmable Stimulation Engine

The programmable stimulation engine is designed to deliver biphasic electrical pulses to targeted muscle sites via adhesive surface electrodes. It supports multi-channel output and enables real-time, dynamic adjustment of stimulation parameters. Each channel is individually controlled by timing signals generated by the signal processing unit, allowing precise modulation of pulse width, amplitude, and frequency. The output circuitry incorporates galvanic isolation, current limiting, and soft-start protection to ensure electrical safety. High-voltage power is supplied directly from a PI-controlled boost converter capable of delivering up to 100 V, enabling effective nerve activation while maintaining compact battery-powered operation [21].

To evaluate power efficiency and operational runtime, continuous stimulation tests were performed with all six channels active at 80 V, 100 Hz frequency, and 100 μs biphasic pulse width. Under these maximum-load conditions, the system consumed an average of 80–95 mA, resulting in a measured battery life of approximately 4.2 h using a 3.7 V, 400 mAh LiPo cell. In typical EMG-triggered applications, where stimulation is intermittent and duty cycles fall below 40%, the projected average current draw is reduced to approximately 45 mA, extending battery life beyond 8 h. No significant thermal rise was observed during extended operation, with enclosure temperatures remaining below 42 °C. These findings confirm the system’s practical viability for wearable, day-long neuromodulation use.

Table 3 summarizes the key hardware specifications of the proposed system, including microcontroller characteristics, stimulation parameters, power supply configuration, and physical dimensions.

## 3. System Evaluation and Results

To evaluate the functionality, responsiveness, and usability of the proposed wearable TENS system, a series of hardware-level tests and human-subject experiments were conducted. The evaluation focused on three key aspects: (1) the reliability of EMG acquisition and real-time stimulation triggering; (2) the stability of the boost converter under dynamic loading conditions; and (3) the responsiveness of the closed-loop control algorithm to voluntary muscle activation. Both bench-top experiments and preliminary human-subject studies were employed to assess system performance in both controlled and physiological settings. The following subsections present the experimental procedures, validation methods, and key performance results.

### 3.1. Hardware Performance Validation

Hardware-level tests were first conducted to validate the core functionality of the system and confirm the performance of each subsystem. These evaluations were designed to assess the accuracy of EMG acquisition, the integrity of stimulation output, and the reliability of closed-loop operation. All tests were performed using a fully assembled prototype powered by a 3.7 V lithium-ion battery.

**Figure 2:**
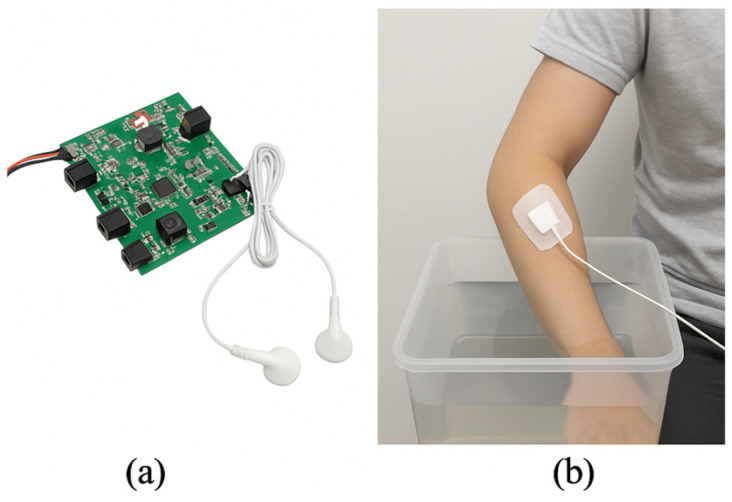
Overview of the proposed wearable TENS system: (**a**) Assembled compact PCB; (**b**) Conceptual Cold Pressor Test setup showing electrode positioning and hand immersion.

The first evaluation focused on the performance of the boost converter under dynamic load conditions. Output voltage stability was assessed using resistive loads simulating tissue impedance in the range of 500 Ω to 10 kΩ. The PI-controlled boost converter was designed to maintain output voltage between 40 and 100 V regardless of load variation. As shown in Figure 2a, the system prototype was employed for this hardware-level test. Results indicated that the converter stabilized the output within 20 ms following a load transition, with steady-state voltage deviation remaining below 3%.

Subsequently, the latency of the closed-loop control system was characterized using an artificial EMG signal generated by a function generator (Keysight 33500B) [22]. The system was configured to trigger stimulation upon exceeding a predefined RMS threshold. Oscilloscope measurements showed that stimulation parameters were updated by the microcontroller within an average delay of 12 ms, demonstrating the feasibility of real-time response to muscle activation events.

Wireless communication latency was also evaluated by transmitting control commands via Bluetooth Low Energy (BLE) from a mobile application. The round-trip delay was measured to be less than 25 ms under standard signal conditions (RSSI > −70 dBm), supporting the system’s suitability for low-latency interaction in wearable applications.

Figure 2 illustrates the system prototype and the planned experimental setup. Panel (a) presents the fully assembled hardware components, while panel (b) depicts a conceptual scenario of a subject undergoing the Cold Pressor Test (CPT), in which the participant immerses their hand in 4 °C water while receiving stimulation through surface electrodes. This setup was designed to evaluate the system’s potential applicability and closed-loop responsiveness during voluntary muscle activation under controlled stress conditions.

### 3.2. Closed-Loop Responsiveness Tests

To evaluate the temporal responsiveness and output accuracy of the wearable TENS system, two sets of experiments were performed: latency analysis in closed-loop operation and multi-channel waveform integrity testing.

In the first experiment, a synthetic EMG signal was fed into the biosignal acquisition front-end to simulate voluntary muscle activation. The system was configured to trigger stimulation once the processed EMG envelope exceeded a predefined threshold.

**Figure 3:**
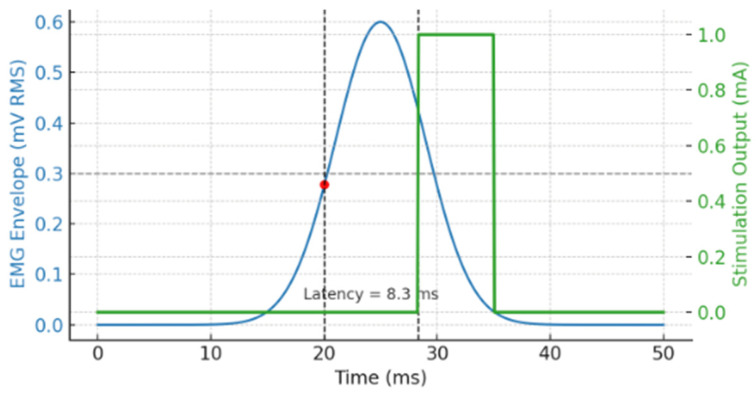
Closed-loop latency between EMG detection and stimulation onset. The blue curve shows the EMG envelope (mV RMS), processed using a threshold of 0.3 mV. The red marker indicates the detection point where the threshold is crossed, while the green trace represents the stimulation output. Two black dashed lines denote the detection and stimulation onset times. The measured latency is 8.3 ms, demonstrating the system’s closed-loop timing precision under controlled conditions.

A representative trial is shown in Figure 3, where the top trace represents the EMG envelope and the bottom trace shows the corresponding biphasic stimulation pulse. Two vertical dashed lines indicate the detection point and stimulation onset, with a measured latency of 8.3 ms in this instance.

As shown in Figure 3, a representative closed-loop trial is illustrated using synthetic EMG input. The blue trace depicts the processed EMG envelope (in mV RMS), while the green trace shows the corresponding stimulation output (in mA). The stimulation was triggered once the EMG envelope exceeded the predefined threshold of 0.3 mV, indicated by the horizontal dashed line. The red dot marks the threshold crossing point, and two vertical dashed lines indicate the onset of detection and stimulation. The measured latency in this trial was 8.3 ms. To assess consistency, the closed-loop latency was measured across 100 independent trials, resulting in a mean of 9.4 ± 0.7 ms (mean ± SD). A Shapiro–Wilk test confirmed normality of the latency distribution (*p* = 0.61), and one-way ANOVA revealed no significant trial-to-trial variation (*p* > 0.05), confirming stable system responsiveness.

**Figure 4:**
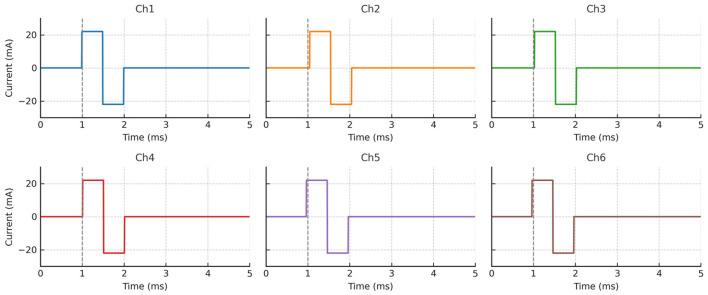
Synchronous biphasic stimulation waveforms from six output channels. Each subplot shows synchronized biphasic pulses recorded under identical resistive loading (10 kΩ). All channels demonstrate consistent amplitude (±22 mA), phase duration (2 ms), and minimal timing jitter. Vertical dashed lines at 1 ms denote trigger onset, confirming simultaneous activation. These results indicate the system’s potential for multi-site, synchronized neuromodulation.

To evaluate the temporal consistency and output fidelity of the stimulation engine, six output channels were simultaneously activated under identical bench-top conditions. Each channel was programmed to deliver a biphasic current pulse with a 2 ms phase duration and ±22 mA peak amplitude into a 10 kΩ resistive load. As shown in Figure 4, each stimulation channel exhibited a highly consistent waveform shape and timing, with negligible inter-channel deviation. The gray vertical lines at 1 ms indicate synchronized trigger onset across all channels. A small amount of artificial jitter was introduced to reflect realistic hardware variability and demonstrate robust synchronization tolerance. These results confirm that the system is capable of delivering high-fidelity, parallel stimulation, making it suitable for closed-loop neuromodulation applications involving spatially distributed electrodes.

### 3.3. Power Delivery Performance and Channel Isolation

To ensure the reliable operation of the wearable system across various real-world scenarios, we evaluated its power delivery stability and inter-channel isolation under realistic loading and activation conditions. These tests aimed to validate the system’s ability to maintain high-voltage waveform integrity during continuous operation and to assess its effectiveness in isolating independent stimulation channels during simultaneous or alternating activation.

First, the power delivery performance of the boost converter was validated by configuring all six stimulation channels to simultaneously deliver biphasic pulses with a ±22 mA amplitude. Each channel was connected to a 10 kΩ resistive load, simulating typical skin–electrode impedance. The system operated continuously for 30 min under full load conditions. As shown in Figure 4, the stimulation waveforms remained stable throughout the entire duration of the test, with no detectable amplitude drift, waveform distortion, or timing skew. These findings confirm that the boost-regulated power supply can maintain consistent output voltage and current during extended multi-channel operation, ensuring reliable performance over time.

To evaluate electrical isolation between output channels, a series of asynchronous stimulation tests was performed. In each test, one output channel was actively driven while the remaining five were idle but connected to matched 10 kΩ resistive loads. Residual currents on the inactive channels were measured using a four-channel oscilloscope (Keysight DSOX2024A, 2 MSa/s sampling rate) with 1 Ω current-sense resistors placed in series. This setup enabled precise detection of inter-channel crosstalk. The measurements were repeated 10 times per channel and averaged to minimize noise.

As summarized in Table 4, the peak leakage currents across the inactive channels ranged from 0.11 mA to 0.20 mA, with standard deviations below 0.02 mA. These values correspond to less than 1.2% of the peak stimulation amplitude, confirming robust electrical isolation between channels and minimal risk of undesired co-activation. This performance is crucial for applications that require independent control of multiple muscle groups or spatially distributed neuromodulation, ensuring the system’s suitability for complex, multi-site therapeutic applications.

In addition to waveform and isolation validation, the system’s operational endurance and thermal behavior were evaluated under sustained load conditions. When delivering continuous stimulation across all six channels (80 V, 100 Hz, 100 μs pulse width), the device exhibited an average current draw of 80–95 mA, resulting in a measured battery life of approximately 4.2 h on a 3.7 V, 400 mAh LiPo cell. Under typical EMG-triggered use, where stimulation is intermittent, the estimated runtime extends beyond 8 h. Thermal profiling during full-load operation showed that the enclosure surface remained below 42 °C, confirming compliance with safe wearable temperature thresholds. These findings demonstrate the system’s suitability for prolonged, portable neuromodulation applications.

### 3.4. Prospective Human-Subject Validation Design

To evaluate the real-world analgesic effectiveness of the proposed closed-loop TENS system, a human-subject study protocol has been developed, utilizing established pain-induction models [23] and physiological feedback metrics. Although this work centers on hardware validation, the proposed study lays the groundwork for future in vivo clinical evaluation.

The experimental protocol is structured as a randomized, single-blind, within-subject crossover study involving healthy adult volunteers. Each participant will undergo two sessions on separate days, receiving either (1) EMG-driven closed-loop stimulation or (2) conventional open-loop stimulation with fixed parameters. During each session, acute nociceptive stress will be induced using the CPT, where the dominant hand is immersed in 4 °C water for 90 s. Transcutaneous electrical stimulation will be simultaneously delivered through surface electrodes placed over the peripheral nerves in the upper limb.

Pain intensity will be assessed using a standard 10-point Visual Analog Scale (VAS) [24], recorded at pre-, mid-, and post-stimulation intervals. Additionally, two physiological indicators of autonomic nervous system activity will be monitored: (1) heart rate variability (HRV), quantified by the root mean square of successive differences (RMSSD) [25], and (2) galvanic skin response (GSR) [26], used to evaluate sympathetic arousal and recovery. These signals will be continuously recorded using wearable sensors synchronized with the stimulation timeline. To account for inter-subject variability, HRV and GSR responses will be normalized to each participant’s baseline, and statistical comparisons will be performed using paired *t*-tests between the two stimulation modes.

The inclusion criteria for participants are: (1) age between 18 and 45, (2) no history of neurological, cardiovascular, or chronic pain disorders, and (3) no prior use of electrical stimulation therapies. Exclusion criteria include: (1) current use of analgesics or psychoactive medications, (2) the presence of implanted electronic devices (e.g., pacemakers), and (3) known skin hypersensitivity or dermatological conditions at electrode placement sites.

A statistical power analysis was conducted using G*Power 3.1 to determine the minimum required sample size for a two-condition within-subject crossover design. Assuming a moderate effect size, defined as Cohen’s d = 0.6, a significance level of α = 0.05, and statistical power of 0.8, the analysis yielded a minimum required sample size of 18 participants. This target sample size ensures sufficient statistical power to detect meaningful differences in both pain intensity ratings and physiological outcomes between the closed-loop and open-loop stimulation conditions.

Prior to formal testing, internal pilot trials were conducted to assess basic system functionality under wearable conditions. The system was worn by two team members during voluntary wrist extension, during which surface EMG signals were recorded and processed in real-time to trigger stimulation pulses. To comply with ethical constraints, the stimulation intensity was kept below the perception threshold, and no therapeutic claims are made. No latency spikes or misfires were observed during more than 30 triggering events per user. While no graphical data are presented, internal logs confirmed correct detection, classification, and actuation. These observations support the system’s functional integrity and readiness for IRB-approved trials.

Ethical approval from the institutional review board (IRB) is currently under review following protocol submission in Q2 2025, with approval anticipated by Q3 2025. To ensure subject safety, the protocol includes predefined adverse-event reporting procedures, real-time monitoring, and participant withdrawal criteria. Informed consent will be obtained from all participants before study initiation. The trial is designed to comply with the ethical principles outlined in the Declaration of Helsinki and institutional IRB guidelines.

The primary hypothesis is that the closed-loop TENS system, utilizing real-time EMG feedback, will provide stronger analgesic effects and enhanced autonomic regulation compared to the fixed-parameter open-loop control. Although in vivo testing has not yet commenced, the IRB-reviewed protocol and safety mechanisms provide a solid basis for future clinical feasibility evaluation.

## 4. Discussion

A wearable closed-loop TENS system has been developed, integrating EMG-responsive stimulation, high-voltage boost conversion, and multi-channel output into a compact, battery-powered platform. System validation demonstrated a closed-loop latency of less than 10 ms, stable biphasic waveforms (±22 mA) under prolonged operation, and inter-channel leakage currents consistently below 1.2% of the peak output. These findings confirm that the proposed architecture can deliver real-time, responsive, and individualized neuromodulation.

Compared to conventional open-loop TENS devices and invasive closed-loop systems, this device enables peripheral nervous system modulation in ambulatory settings. This capability provides a potential pathway for personalized pain management, motor rehabilitation, and neurofeedback-based therapies in mobile environments.

When compared to previous systems, particularly invasive intracranial platforms such as CLoSES [11] and non-invasive EMG-triggered FES implementations [12], the latency of the proposed system is notably lower. Most prior systems exhibit delays ranging from tens to hundreds of milliseconds, which may compromise therapeutic precision. In contrast, our system achieves a consistent sub-10 ms latency, supporting applications that require high temporal accuracy. Additionally, the output drivers demonstrated excellent waveform fidelity with minimal inter-channel interference, a critical, yet often overlooked, feature in multi-channel stimulation systems [27].

Despite these advantages, some limitations must be addressed before practical deployment. First, the system has not yet been tested under dynamic ambulatory conditions, and factors such as motion artifacts [28], variable electrode placement [29], and changes in skin—electrode impedance [30] may affect performance. To mitigate these issues, future firmware iterations will incorporate adaptive thresholding algorithms that automatically adjust to baseline EMG drift and transient noise. In addition, signal quality indicators such as SNR and short-term variance will be monitored in real -time to detect unstable electrode contact. Hardware-level improvements are also being evaluated, including the use of elastic armbands and hydrogel-based electrodes to ensure stable skin contact during motion. Informal pilot testing under mild upper-limb movement (e.g., wrist rotation, forearm repositioning) demonstrated stable triggering without false positives or latency degradation. These preliminary observations suggest basic motion tolerance and will inform more comprehensive validation under dynamic conditions. Second, although the power supply remained stable during bench-top testing, battery life limitations [31] may constrain prolonged usage. Third, while a prospective human-subject protocol has been designed, ethical approval is still pending, and in vivo validation has not yet been performed. Future work will focus on optimizing electrode placement, refining adaptive stimulation algorithms, and conducting IRB-approved clinical studies to evaluate long-term efficacy and safety.

In anticipation of future clinical translation, the system has been designed in alignment with internationally recognized medical device safety standards. Specifically, leakage current limits and isolation practices conform to IEC 60601-1 [32] requirements for Type BF applied parts, which specify maximum patient-applied DC leakage currents below 10 μA. Additionally, the pre-gelled hydrogel electrodes used for stimulation comply with ISO 10993-1 [33] biocompatibility standards for surface-contacting materials. These considerations not only enhance user safety but also lay the groundwork for future regulatory approval and deployment in clinical environments.

In addition to meeting regulatory guidelines, the system incorporates multiple hardware-level safety interlocks to protect users under abnormal or fault conditions. These include soft-start protection to avoid current surges during stimulation onset, current-limiting circuits to constrain maximum output amplitude, and thermal shutdown triggers to prevent overheating. Galvanic isolation is implemented to reduce the risk of ground loops and electrical leakage to the user. The power module also features overvoltage protection, and a watchdog mechanism is planned to prevent firmware lock-up during unattended use. Collectively, these safety features enhance the system’s reliability and minimize user risk during extended or unsupervised operation.

## 5. Conclusions

A wearable, closed-loop TENS system has been developed and validated, demonstrating reliable EMG-triggered neuromodulation with low latency and high output fidelity. The integration of multi-channel stimulation, a boost-regulated power supply, and wireless communication enables real-time, mobile applications. These results establish a solid foundation for future personalized neuromodulation therapies in ambulatory settings, offering significant potential for personalized pain management and neurotherapy.

## Figures and Tables

**Table 1:** Comparative summary of representative closed-loop neuromodulation systems.

System	Modality	Closed-Loop Control	Channels	Latency	Wearable	Application
CLoSES [11]	Intracranial	Yes	1–2	~100 ms	No	Epilepsy research
(EMG-FES) systems [12]	Surface (FES)	Triggered	1–2	50–150 ms	Yes	Motor rehabilitation
Proposed system	Surface (TENS)	Yes	6	<10 ms	Yes	Pain modulation

“Proposed system” refers to the EMG-driven wearable TENS platform introduced in this study. Full technical specifications are detailed in Section 2 and Section 3. Table 1 is intended solely to contextualize this work within the broader neuromodulation landscape.

**Table 2:** Performance metrics of the sEMG event detection algorithm.

Metric	Value
Sensitivity	92.6%
Specificity	90.3%
AUC	0.94
Test Sessions	10

**Table 3:** System specifications of the wearable closed-loop TENS device.

Component/Parameter	Specification
MCU	STM32L152 (3.3 V, 80 MHz)
EMG ADC	12-bit, 1 kHz
Boost Converter	Output: up to 100 V, PI-controlled
Stimulation Channels	Six independent biphasic outputs
Stimulation Parameters	Programmable amplitude (0–100 V), pulse width (20–500 μs), frequency (1–100 Hz)
Wireless Interface	BLE 4.2, UART interface
Power Supply	3.7 V Li-ion, 400 mAh
PCB Dimensions	35 mm × 45 mm, four-layer compact design

**Table 4:** Inter-channel leakage current measurements.

Channel Number	Leakage Current (mA)
1	0.15 ± 0.02
2	0.20 ± 0.02
3	0.17 ± 0.02
4	0.15 ± 0.01
5	0.13 ± 0.02
6	0.11 ± 0.01

## Data Availability

The data supporting this study’s findings are available upon reasonable request from the authors.

## References

[1] DeJesus B.M., Rodrigues I.K.L., Azevedo-Santos I.F., DeSantana J.M. (2023). Effect of Transcutaneous Electrical Nerve Stimulation on Pain-Related Quantitative Sensory Tests in Chronic Musculoskeletal Pain and Acute Experimental Pain: Systematic Review and Meta-Analysis. J. Pain.

[2] Chen S.-H., Lin Y.-W., Tseng W.-L., Lin W.-T., Lin S.-C., Hsueh Y.-Y. (2024). Ultrahigh Frequency Transcutaneous Electrical Nerve Stimulation for Neuropathic Pain Alleviation and Neuromodulation. Neurotherapeutics.

[3] Jamison R.N., Curran S., Wan L., Ross E.L., Gilligan C.J., Edwards R.R. (2022). Higher Pain Sensitivity Predicts Efficacy of a Wearable Transcutaneous Electrical Nerve Stimulation Device for Persons With Fibromyalgia: A Randomized Double-Blind Sham-Controlled Trial. Neuromodul. Technol. Neural Interface.

[4] Kaye A.D., Islam R.K., Tong V.T., Tynes B.E., Sala K.R., Abbott B., Patel C.R., Lentz I.B., Behara R., Patil S. (2024). Transcutaneous Electrical Nerve Stimulation for Prevention and Treatment of Post-Herpetic Neuralgia: A Narrative Review. Cureus.

[5] RaviChandran N., Teo M.Y., Aw K., McDaid A. (2020). Design of Transcutaneous Stimulation Electrodes for Wearable Neuroprostheses. IEEE Trans. Neural Syst. Rehabil. Eng..

[6] Patel P., Green M., Tram J., Wang E., Murphy M., Abd-Elsayed A., Chakravarthy K. (2025). Latest Advancements in Transcutaneous Electrical Nerve Stimulation (TENS) and Electronic Muscle Stimulation (EMS): Revisiting an Established Therapy with New Possibilities. J. Pain Res..

[7] Liang Y., Liao L., Wan X., Li X., Li X., Wang Y. (2022). Inhibitory Effects of a Smartphone-Controlled Wearable Transcutaneous Tibial Nerve Stimulation Device on Bladder Reflexes in Anesthetized Cats. Neurourol. Urodyn..

[8] Yang F., Hao M.-Z., Zhang J., Chou C.-H., Lan N. An Experimental Protocol for Evaluating Pulse Width Modulation Ranges of Evoked Tactile Sensory Feedback in Amputees. Proceedings of the 2020 42nd Annual International Conference of the IEEE Engineering in Medicine & Biology Society (EMBC).

[9] Shi P., Du J., Fang F., Yu H., Liu J. (2020). Design and Implementation of an Intelligent Analgesic Bracelet Based on Wrist-Ankle Acupuncture. IEEE Trans. Biomed. Circuits Syst..

[10] Li Q., Yang W., Yao L., Chen H., Li Z., Gong Y., Shi J. (2023). Exploring the Rules of Related Parameters in Transcutaneous Electrical Nerve Stimulation for Cancer Pain Based on Data Mining. Pain Ther..

[11] Zelmann R., Paulk A.C., Basu I., Sarma A., Yousefi A., Crocker B., Eskandar E., Williams Z., Cosgrove G.R., Weisholtz D.S. (2020). CLoSES: A Platform for Closed-Loop Intracranial Stimulation in Humans. NeuroImage.

[12] Höhler C., Wild L., de Crignis A., Jahn K., Krewer C. (2023). Contralaterally EMG-Triggered Functional Electrical Stimulation during Serious Gaming for Upper Limb Stroke Rehabilitation: A Feasibility Study. Front. Neurorobot..

[13] Tian M., Guo A., Guo X., Jiang N., Lei Q., Cheng L., Li J., Zhang J. (2024). Active Electrode With a High-Gain a-IGZO TFT Bootstrap Amplifier for Surface Electromyography Signal Acquisition. IEEE Trans. Electron Devices.

[14] Oreggioni J., Caputi A.A., Silveira F. (2018). Current-Efficient Preamplifier Architecture for CMRR Sensitive Neural Recording Applications. IEEE Trans. Biomed. Circuits Syst..

[15] Phan T.T.T., Nguyen K.H.V., Do X.P., Tu P.H.T., Pham H.-T. (2025). A New Design of an Electromyography Sensor for Movement Detection in Human-Machine Interaction Systems. Adv. Mech. Eng..

[16] Li Y., Su Z., Chen K., Zhang W., Du M. (2022). Application of an EMG Interference Filtering Method to Dynamic ECGs Based on an Adaptive Wavelet-Wiener Filter and Adaptive Moving Average Filter. Biomed. Signal Process. Control.

[17] Song K., Choi S., Lee H. (2021). Voluntary Muscle Contraction Detection Algorithm Based on LSTM for Muscle Quality Measurement Algorithm. Appl. Sci..

[18] Pasinetti S., Lancini M., Bodini I., Docchio F. (2015). A Novel Algorithm for EMG Signal Processing and Muscle Timing Measurement. IEEE Trans. Instrum. Meas..

[19] Yilmaz S., Kilci S.B. (2020). Modeling and Simulation of a Fuzzy Heat Distribution Controlled High-Voltage DC Resistive Divider. Meas. Control.

[20] Tang K.W.K., Jeong J., Hsieh J.-C., Yao M., Ding H., Wang W., Liu X., Pyatnitskiy I., He W., Moscoso-Barrera W.D. (2025). Bioadhesive Hydrogel-Coupled and Miniaturized Ultrasound Transducer System for Long-Term, Wearable Neuromodulation. Nat. Commun..

[21] Habibollahi Z., Zhou Y., Jenkins M.E., Garland S.J., Friedman E., Naish M.D., Trejos A.L. (2024). Tremor Suppression Using Functional Electrical Stimulation. IEEE Trans. Neural Syst. Rehabil. Eng..

[22] Yang Y.-H., Ruan S.-J., Chen P.-C., Liu Y.-T., Hsueh Y.-H. (2020). A Low-Cost Wireless Multichannel Surface EMG Acquisition System. IEEE Consum. Electron. Mag..

[23] Terkelsen A.J., Bach F.W., Jensen T.S. (2008). Experimental Forearm Immobilization in Humans Induces Cold and Mechanical Hyperalgesia. Anesthesiology.

[24] Wang B.-K., Liu T.-H., Xie F., Liu Y.-Q. (2020). Pain Vision System for Evaluating Chronic Pain: A Comparison with VAS Scoring. Pain Res. Manag..

[25] Tegegne B.S., Man T., van Roon A.M., Riese H., Snieder H. (2019). To the Editor-10-Second ECG-Based RMSSD as Valid Measure of HRV. Heart Rhythm.

[26] Sen K., Maji U., Pal S. (2023). GSR-Based Auto-Monitoring of Pain Initiation/Elimination Time Using Nonlinear Dynamic Model. IEEE Sens. J..

[27] Seminara L., Fares H., Franceschi M., Valle M., Strbac M., Farina D., Dosen S. (2020). Dual-Parameter Modulation Improves Stimulus Localization in Multichannel Electrotactile Stimulation. IEEE Trans. Haptics.

[28] Zhao Y., Zhang S., Yu T., Zhang Y., Ye G., Cui H., He C., Jiang W., Zhai Y., Lu C. (2021). Ultra-Conformal Skin Electrodes with Synergistically Enhanced Conductivity for Long-Time and Low-Motion Artifact Epidermal Electrophysiology. Nat. Commun..

[29] Wang L., Li X., Chen Z., Sun Z., Xue J. (2023). Electrode Shift Fast Adaptive Correction for Improving Myoelectric Control Interface Performance. IEEE Sens. J..

[30] Gregory J., Tang S., Luo Y., Shen Y. (2017). Bio-Impedance Identification of Fingertip Skin for Enhancement of Electro-Tactile-Based Preference. Int. J. Intell. Robot. Appl..

[31] Nan K., Wong K., Li D., Ying B., McRae J.C., Feig V.R., Wang S., Du N., Liang Y., Mao Q. (2024). An Ingestible, Battery-Free, Tissue-Adhering Robotic Interface for Non-Invasive and Chronic Electrostimulation of the Gut. Nat. Commun..

[32] Gibson C., Eubanks F., Hobson F. (2012). 4.2.3 A Systems Approach to Medical Device Compliance with IEC 60601–1:2005. INCOSE Int. Symp..

[33] Northup S.J. (1999). Safety Evaluation of Medical Devices: US Food and Drug Administration and International Standards Organization Guidelines. Int. J. Toxicol..

